# Ultrasound-assisted deep eutectic solvent extraction of polyphenols from *Cornus officinalis*: Optimization, mechanisms, and bioactivity

**DOI:** 10.1016/j.ultsonch.2026.107909

**Published:** 2026-06-01

**Authors:** Peng Chen, Wenhao Xiao, Ruixiang Li, Benhong Zhou, Fuchao Chen, Ruhong Zhang

**Affiliations:** a Department of Pharmacy, Renmin Hospital of Wuhan University, Wuhan, Hubei 430060, PR China; b Wuhan University School of Pharmaceutical Sciences, Key Laboratory of Combinatorial Biosynthesis and Drug Discovery, Ministry of Education, China; c Department of Psychiatry of Wuhan Wudong Hospital, Wuhan, Hubei 430085, PR China; d Sinopharm Dongfeng General Hospital, Hubei University of Medicine, Shiyan, Hubei 442001, PR China; e Hubei Key Laboratory of Wudang Local Chinese Medicine Research, School of Pharmaceutical Sciences, Hubei University of Medicine, Shiyan, Hubei 442000, China; f Health Management Center, Renmin Hospital of Wuhan University, Wuhan, Hubei 430060, PR China

**Keywords:** Cornus officinalis, Polyphenols, Deep eutectic solvents, Ultrasonic, Bioactivity, Molecular dynamic simulation

## Abstract

This study investigated the combined use of deep eutectic solvents (DESs) and ultrasound-assisted extraction (UAE) for extracting polyphenols from *Cornus officinalis* (COF). The results revealed that DESs containing choline chloride–acetamide (ChCl–Ace, 1 : 2) yielded the highest extraction efficiency. Optimal extraction conditions included an ultrasonic power of 350 W, a liquid-to-solid ratio of 24 : 1 mL/g, and 45% water content. Eight phenolic compounds in the COF extract were identified using high-performance liquid chromatography–mass spectrometry. According to the SEM observations, the surface structure of the samples was modified by UAE, as indicated by an average pore size of 770 ± 14.32 nm in ChCl–Ace–UAE, which helped release phenolic compounds. Molecular dynamics simulations showed that ChCl–Ace significantly enhanced solute–solvent interactions, particularly with gallic acid and cyanidin-3-O glucoside, leading to improved extraction efficiency. *In vitro* chemical and cellular assays confirmed that DES–Ace displayed superior antioxidant properties. In addition, the ChCl–Ace–UAE extract (0.5 mg/mL) demonstrated 91.25 ± 0.15% *Staphylococcus aureus*, 95.48 ± 0.17% *Escherichia coli* and 93.72 ± 0.36% *Propionibacterium acnes* inhibition in the antibacterial assay. In summary, the combined use of UAE and DESs offers a promising and eco-friendly approach for extracting valuable compounds from botanical sources.

## Graphical abstract

**Figure:**
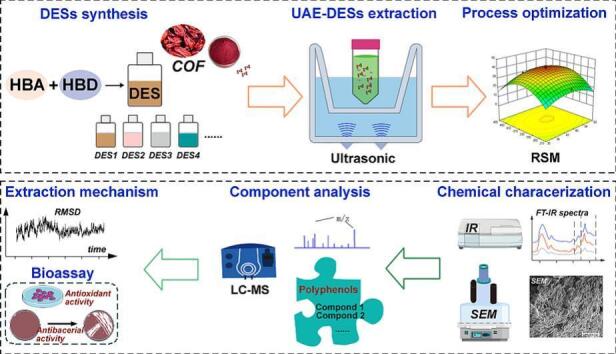

## 1 Introduction

Polyphenols are a large and heterogeneous family of bioactive molecules found in numerous plants such as fruits, vegetables, herbs, spices, and tea [1]. Research indicates that plant-based diets abundant in polyphenols can confer health benefits by mitigating the risk of various diseases, including cancer as well as neurodegenerative, cardiovascular, and inflammatory conditions [2]. Consequently, investigating novel sources of bioactive plant polyphenols and conducting comprehensive characterization is essential to enhance human health.

*Cornus officinalis* (COF, known as Shanzhuyu in Chinese), a deciduous shrub or small tree from the Cornaceae family, is known for its fruits, rich in phenolic compounds [3]. These compounds enhance immune function, lower blood glucose levels, and demonstrate antioxidant properties, suggesting that COF has good potential for development and utilization as a functional food and nutraceutical [4]. Nevertheless, the applications of COF and its bioactive compounds in the food and functional food industries remain limited. Therefore, to promote the potential application of COF as a healthy value-added product in the functional food industry, establishing environmentally friendly and efficient approaches for the extraction and preparation of bioactive polyphenols from COF is necessary [5].

Currently, the conventional solvent extraction (CSE) method is commonly used to extract phenolic compounds. However, these methods have disadvantages such as high energy consumption, low efficiency, long extraction times, and increased pollution levels [6], [7]. Consequently, the scientific community is increasingly focusing on deep eutectic solvents (DESs) due to their potential as cost-effective and environmentally sustainable alternative [8]. The utilization of DESs enables the effective extraction of phenolic compounds from raw materials, eliminating the necessity for organic solvents [9]. These solvents are clear and homogeneous liquids formed by thermally combining hydrogen bond donors (HBDs) and hydrogen bond acceptors (HBAs) at specific molar ratios [10]. For instance, Lin et al. utilized DESs containing choline chloride and fruit acid (ChCl–Fru) for extracting polyphenols from quinoa, demonstrating that the DESs extraction method surpassed ethanol extraction in terms of efficiency [11]. Similarly, Linyang Wang and his colleagues used DESs formulated from betaine and lactic acid to extract polyphenols from *Anchusa italica Retz* flowers. This indicates that the ultrasound-assisted DESs extraction method provides notable benefits in efficiency and sustainability [12].

The utilization of ultrasound-assisted extraction (UAE) for extracting natural plant materials is on the rise due to its high efficiency, ease of use, and environmentally friendly characteristics [13]. Ultrasonic technology enhances the interaction between the solvent and solute by promoting material transfer and increasing surface contact, including disrupting plant cell walls, thereby enhancing extraction efficiency [14]. The ultrasonic power plays a crucial role in this process. Increasing the ultrasonic power can boost both energy output and cavitation intensity, thereby accelerating material transfer and enhancing extraction efficiency [15]. Several studies have demonstrated that integrating ultrasound into DESs leads to a significant assisted effect, enhancing the efficiency of extracting bioactive compounds from natural sources [16]. Consequently, the combined application of UAE and DESs presents a promising approach for the environmentally sustainable and efficient extraction of phenolic compounds from COF. However, the potential of ultrasound-assisted DESs for isolating bioactive phenolic compounds from COF remains unexplored.

The aim of this study was to evaluate a UAE technique assisted by DESs for the efficient extraction of phenolic compounds from COF. The extraction process was optimized through response surface methodology (RSM). Molecular dynamics (MD) analysis was used to investigate the interactions between the primary phenolics and various solvents. Additionally, a comparative assessment of the chemical composition, antioxidant activity, and antibacterial properties of COF extraction was carried out using both the UAE-assisted DESs method and conventional ethanol extraction methods.

## 2 Materials and methods

### 2.1 Materials and chemicals

Choline chloride, glycol, glycerol, D-mannitol, N-butanol, malonate, formic acid, oxalic acid, lactic acid, levulinic acid, citric acid, glucose, urea, methylurea, acetamide, glycine, Folin–Ciocalteu, 2,4,6-tris (2-pyridyl)-s-triazine (TPTZ), 1,1-diphenyl-2-picryl-hydrazyl hydrate (DPPH) and 2,2′-azino-bis (3- ethylbenzo −thiazoline-6-sulfonic acid) diammonium salt (ABTS) were purchased from Thermo Fisher Scientific (Massachusetts, America) Company. Ethanol, methanol and other reagents were provided by China National Medicines Corporation Ltd. (Beijing, China). All reagents used in this experiment were of analytical grade. COF was obtained from the Jiangxi Jiangzhong Prepared Slices of Chinese Crude Drugs Co Ltd. The fruits underwent a thorough wash, and cellulose gauze was employed to get rid of the surplus water and they were store in a refrigerator at 4 ℃.

### 2.2 Dess preparation

The DESs were prepared following the method described by Li et al. [17], with the amounts of HBD and HBA components used listed in Table S1. To produce a clear and homogeneous liquid known as DESs, the solution was immersed in water at 80 °C for 30 to 120 min. Subsequently, the mixture was allowed to cool to 25 °C naturally. Consistent with established protocols, the physicochemical properties such as pH, viscosity, and polarity were examined [18].

Before extracting, impurities were removed from the COF pulp, followed by drying the material at a temperature of 50 °C. The dried material was then finely ground and sieved through a 20-mesh sieve. A 0.5 g sample of dried COF powder was combined with 5 mL of various DESs, methanol, 65% methanol, ethanol, and 65% ethanol, followed by ultrasonic extraction at 50 °C for 50 min with a power of 320 W. The extract was centrifuged at 10,000 × g for 20 min, and the resulting supernatant was promptly collected and stored at 4 °C for subsequent analysis.

### 2.3 Determination of total phenolic and anthocyanin contents in COF

As outlined in previous study [19], the total phenolic content (TPC) of COF was determined using the Folin–Ciocalteu method. Using gallic acid for standardization, the findings were reported as milligrams of gallic acid equivalents per gram of dry herbal material. The total anthocyanin content (TAC) was assessed using an adapted spectrophotometric technique described by Che et al. (2024) [7]. Potassium chloride (0.025 mol/L) was used to prepare a pH = 1 buffer solution, while sodium acetate (0.5 mol/L) was used for a pH = 4.5 buffer. The solution was then incubated in the dark for 15 min at room temperature. Absorbance was recorded at wavelengths of 510 and 700 nm. Equations (a) and (b) were used to calculate the TAC, calculated as the cyanidin-3-O-glucoside equivalent per gram of dry weight of COF (mg CE/g DW).

(a) $$A\;=\left[A510-A700\right]pH1.0-\left[A510-A700\right]pH4.5$$

$$\mathrm{TAC}=\frac{A*Mw*DF}{\varepsilon l}1000$$

where *A* represents the absorbance value obtained from the equation, *Mw* is an abbreviation for the molecular weight of anthocyanin, *DF* is the dilution factor, *ε* represents the extraction coefficient, with a molar absorptivity of 26,900 L/cm·mol and a path length of 1 cm.

### 2.4 Single-factor optimization test

To ensure the feasibility of the experimental procedure, five main factors were investigated. The influence of five independent variables on the polyphenol concentration was examined using a single-factor experimental design. The variables studied included ultrasonic temperature (30, 40, 50, 60, and 70 °C), ultrasonic duration (15, 20, 25, 30, and 35 min), ultrasonic power (200, 240, 280, 320, and 360 W), liquid-to-solid ratio (3:1, 4:1, 5:1, 6:1, and 7:1 mL/g), and the amount of water added (10, 20, 30, 40, and 50%) [12]. A precooled circulating water bath combined with ice cubes was used to mitigate temperature fluctuations caused by ultrasonic cavitation [11]. The water bath was precooled with ice before sonication, and circulation was maintained to ensure a uniform temperature. Ice cubes were added as required during the experiment to stabilize the system temperature and ensure reproducible conditions.

### 2.5 Optimization of extraction conditions for ChCl–Ace–UAE

Based on the extraction yield results, the DESs demonstrating the highest extraction efficiency were chosen for optimizing the extraction procedure. Prior to implementing the RSM, the key influencing factors, specifically the extraction temperature, ultrasonic duration, ultrasonic power, water addition, and liquid-to-solid ratio, were evaluated through single-factor experiments. The outcomes of these experiments indicated that ultrasound power levels of 200, 280, 360, 420, 500, and 580 W, water additions of 15%, 30%, 45%, 65%, and 85%, and liquid-to-solid ratios of 6:1, 12:1, 18:1, 24:1, 30:1, and 36:1 mL/g were appropriate for further analysis using RSM [11], [12]. Subsequently, Design-Expert [13] software from Stat-Ease was utilized to employ a three-factor, three-level Box-Behnken design.

Second-order polynomial equations were used to demonstrate the relationship between the independent variables and the response variables, YTPC and YTAC, as depicted in the following equation:

$$Y=\beta_{0}+\sum_{i=1}^{n}\beta_{i}X_{i}+\sum_{i=1}^{n}\sum_{j=i+1}^{n-1}\beta_{\mathrm{ij}}X_{i}X_{j}+\sum_{i=1}^{n}\beta_{\mathrm{ii}}X_{i}^{2}$$

where Y refers to the response variables, which are TPC and TAC, the intercept of the model is β_0_, and the coefficients for linear, squared, and interaction terms are represented by β_i_, β_ii_, and β_ij_. The variables X_i_ and X_j_ do not depend on each other, and n represents the number of variables examined, which is three.

### 2.6 FTIR analysis

The sample underwent a 48-h freeze-drying process at −80 °C in a vacuum using a freeze dryer made in Wuhan, China. Following this, the dried sample was combined with potassium bromide and shaped into a pellet. The FTIR spectra were obtained using the ReactIR™ Spectrometer (METTLER TOLEDO, Switzerland). Spectroscopic analysis was conducted in transmission mode within the wavenumber range of 400–4000 cm^−1^.

Quantitative analyses, such as deconvolution of the O–H band, were performed to compare different hydrogen-bonding populations as previously described [20]. The broad O–H stretching vibration peak at 3200–3600 cm^−1^ was selected, and peak fitting was performed using the Origin 2024 software. Baseline correction was performed using the adaptive least squares method (AsLS), and the Savitzky–Golay filter (window of seven points) was used for smoothing and noise reduction. The second-derivative method was employed to locate the peak positions. The Gaussian function was used to separate the hydroxyl peaks, and the fitting algorithm was the Levenberg–Marquardt least squares method, with a fitting goodness of R^2^ ≥ 0.9998. The hydroxyl peaks were separated into 3 categories: free hydroxyl (FH) at 3570–3590 cm^−1^, strongly bound hydroxyl (SBH) at 3420–3440 cm^−1^, and weakly bound hydroxyl (WBH) at 3270–3290 cm^−1^. The area proportions of each subpeak were calculated, and the hydrogen bond network characteristics of the different systems were analyzed.

### 2.7 HPLC-ESI-QTOF-MS/MS analysis

HPLC and electrospray ionization–quadrupole time-of-flight–mass spectrometry (ESI-qTOF-MS) techniques were utilized to analyze the pulp from the COF. The polyphenol extraction samples designated for analysis were freeze-dried and then reconstituted with 6 mL of chromatographic-grade methanol in a specified proportion. The chromatographic analysis was conducted using an Inertsil ODS-3 reverse-phase column (Shimadzu, Japan, 150 × 4.6 mm, 3 µm). Phase A comprised water with 0.1% formic acid, while phase B comprised pure methanol. Each sample was filtered using a 0.45 μm membrane filter, following which 3 μL was injected. The elution profile was as follows: 10% A and 10% B from 0 to 2 min; 10% A and 20% B from 2 to 8 min; 20% A and 35% B from 8 to 37 min; 35% A and 40% B from 37 to 53 min; 40% A and 63% B from 53 to 64 min; 63% A and 30% B from 64 to 66 min; 30% A and 5% B from 66 to 68 min; and 5% A and 5% B from 68 to 75 min. The mobile phase was maintained at a flow rate of 0.5 mL/min. The column temperature was set at 30 °C, and the detection wavelengths were 280 nm and 350 nm.

A mass-to-charge ratio range of 150–2000 was employed to obtain full-scan mass spectra in the negative ionization mode. In ESI-qTOF-MS/MS analysis, the operational parameters comprised a −15 V capillary voltage, a 4 kV source voltage, a capillary temperature of 220 °C, and a sheath gas flow rate of 30 A.U. The tentative identification of bioactive compounds was conducted by observing *m*/*z* values, comparing retention times (RT), and fragment ion patterns according to commercial standards and published reference [21].

### 2.8 Scanning electron microscopy

The structural characteristics of all samples were evaluated by analyzing the morphology of the COF residue using scanning electron microscopy (SEM) (HITACHI, Japan) to investigate the impact of various solvents. Approximately 5 mg of the COF residue was freeze-dried and then mounted on a sample holder for SEM analysis. Subsequently, the COF residue was placed in the SEM chamber for surface morphological examination via electron beam scanning. An accelerating voltage of 5 kV was applied for imaging. Quantitative analysis of the SEM images was conducted using Image J software. The equivalent circular diameter (ECD) was used to calculate the pore diameter, and the average pore size was statistically determined.

### 2.9 MD simulations

The Amber ff99SB force field and the GROMACS software package (version 2020) were used to conduct the MD simulations [22]. PubMed provides the structures of important molecules, such as cyanidin-3-O-glucoside (C3G) and gallic acid (GA). ORCA software was used to generate the structures of ethanol and ChCl-Ace, which were optimized using density functional theory at the B3LYP/6-311G** level to obtain the configurations with the lowest energy [23]. MultiFwn software was used to compute the restricted electrostatic potential charge [24]. Topological parameters from the ACPYPE web tool were used to construct a simulation system for these molecules [25].

The Packmol software was utilized to set up three systems, each enclosed in a cubic box measuring 55 Å × 55 Å × 55 Å: (1) C3G or GA and ChCl–Ace (C3G–ChCl–Ace or GA–ChCl–Ace) system, (2) C3G or GA and 65% ethanol (C3G–65% EtOH or GA–65% EtOH) system [26]. A fixed distance of 1.2 nm was employed to truncate the van der Waals and short-range Coulomb interactions, with the particle mesh Ewald method utilized for computing the long-range Coulomb interactions [27]. To manage the temperature and pressure of the system, the V-rescale and Berendsen barostat algorithms were applied over the duration of 0–300 ps. Finally, a 100 ns simulation was conducted within the NPT ensemble, utilizing a 2 fs time step. The details for the composition of simulated system is presented in Table 1.

**Table 1:** The details for the composition of simulated system.

No.	Companion system (solute–solvent)	Solute molecules	Solvent molecules	Box dimensionsafter equilibration (nm)	Final density (g/cm^3^)
1	C3G-65%EtOH	8	7908	4.80 × 4.80 × 4.8	0.91
2	C3G-ChCl-Ace	4	864	5.25 × 5.25 × 5.25	1.072
3	GA-65%EtOH	16	7914	4.0 × 4.0 × 4.0	0.94
4	GA-ChCl-Ace	8	832	5.0 × 5.0 × 5.0	1.07

### 2.10 Assessment of antioxidant activity *in vitro via* chemical methods

To evaluate the *in vitro* antioxidant activities, four chemical approaches were implemented: scavenging of DPPH and ABTS^+^ radicals, ferric reducing antioxidant power (FRAP), and reducing activity (RA) [28]. The radical scavenging activity of DPPH and ABTS + was measured based on the equations. Trolox was used as a reference for measuring the radical-scavenging activity. The results were quantified as micromoles of Trolox equivalents per gram of COF dry weight. FRAP and RA assays were performed according to the procedure described by Che et al7. In the FRAP assay, a standard curve was developed using FeSO_4_·7H_2_O (0–1000 μM) as a reference, resulting in an R^2^ value of 0.9999. The results were reported in terms of millimoles of ferrous sulfate equivalents per gram of dry weight sample (mM Fe(II)SE/g DW). RA was quantified in micromoles of Trolox equivalents per gram of the dry sample [28]. Concentrations from 100 to 800 Âµmol per milliliter were used to construct Trolox calibration curves, which achieved an R^2^ of 0.9989. Each assay was conducted in triplicate.

The radical scavenging activity against DPPH or ABTS^+^ was calculated as a percentage using the following equation:

$$=\;\left[1\;-\;\left(C1-C2\right)/C0\right]\;\times \;100$$

where C0 represents the absorbance of the reaction solution upon mixing with the PBS solution, while C1 indicates the absorbance measured when the reaction solution interacts with the sample solution, and C2 denotes the absorbance of the sample solution.

### 2.11 Evaluation of cell viability, MDA level, and antioxidant enzyme activities (CAT, GSH-Px, and SOD) in the RAW264.7 cells

The experimental methods for RAW264.7 cell culture and antioxidant capacity determination were detailed by Siddiqui et al. [29]. RAW264.7 cells, in the logarithmic growth phase, were treated with 0.25% trypsin to yield a suspension at a concentration of 5 × 10^5^ cells/mL in complete DMEM. These cells were then seeded into 96-well plates and exposed to extracts at various concentrations dissolved in complete medium. After a 24-h attachment period, DES-derived extracts were applied at concentrations of 0.1, 0.25, and 0.5 mg/mL (respectively containing 226 ± 7.16, 435 ± 12.06, and 861 ± 19.35 μg GAE/g extract). Following an additional 24 h of incubation, the supernatant was removed, and 150 μL of MTT solution (1 mg/mL) was added to each well. Cell viability was determined after a further 4-hour incubation, with each concentration evaluated in triplicate.

For the analysis of oxidative stress, RAW264.7 cells were treated with H_2_O_2_ after a 24-h period of extract treatment. The cells were washed with PBS after the removal of the supernatant prior to protein extraction. Kits (Beyotime) were used to measure the levels of catalase (CAT), glutathione peroxidase (GSH-Px), superoxide dismutase (SOD), and malondialdehyde (MDA), following the instructions provided by the manufacturer’s instructions. The same procedure was repeated thrice.

### 2.12 Evaluation of antibacterial effects

The colony counting method was utilized to evaluate the antibacterial effectiveness of the COF extracts [30]. The process of preparing bacterial cultures began by retrieving the frozen *Staphylococcus aureus* stock from storage at −80 °C. A sterile inoculation loop was used for rapid thawing, followed by aseptic streaking onto a blood culture dish known as N1 plates. After an incubation period of 24 h at 37 °C, individual colonies were chosen from the N1 plates and transferred to N2 plates to ensure culture purity. The N2 plate was then further incubated for 24 h under the same conditions to obtain a sterile bacterial culture.

In subsequent experiments, a bacterial suspension with a concentration of 1 × 10^6^ CFU/mL was prepared by homogenizing the colonies on an N2 plate in a sterile 0.85% NaCl solution. Simultaneously, a specified volume (X mL) of the UV-sterilized solution was added to the bacterial suspension in a sterile test tube. A bacterial suspension without additives served as the blank control. Incubation of all experimental and control groups occurred at 37 °C for 12 h, with a constant agitation speed of 100 rpm to maintain consistent interaction conditions. The bacterial suspensions underwent a 1:1000 serial dilution after the incubation period. Newly prepared blood culture dishes were evenly coated with 50 μL aliquots from the diluted suspensions. The plates were then incubated at 37 °C for 24 h to determine the number of living bacteria. Following incubation, the colonies were photographed and counted. All experiments were performed in triplicate. Finally, the formula was utilized to calculate the bactericidal effectiveness of each membrane.

$$\text{Bactericidal Rate}\left(\%\right)=1-\;\frac{\text{CFU}\;\text{experimental}}{\text{CFU blank}}$$

In the experimental and control groups, colony counts are denoted by CFU_experiment_ and CFU_blank_, respectively.

### 2.13 Statistical analysis

GraphPad Prism 8.0 software, developed by GraphPad Software in the USA, was utilized for statistical analysis and graphics. One-way ANOVA and Tukey's HSD test were applied to analyze the experimental data to assess significant differences among the groups. Statistical significance was denoted by a p-value less than 0.05.

## 3 Results and discussion

### 3.1 Effect of DES type on phenolic and anthocyanin extraction

#### 3.1.1 Selection of optimal DES system

**Fig. 1:**
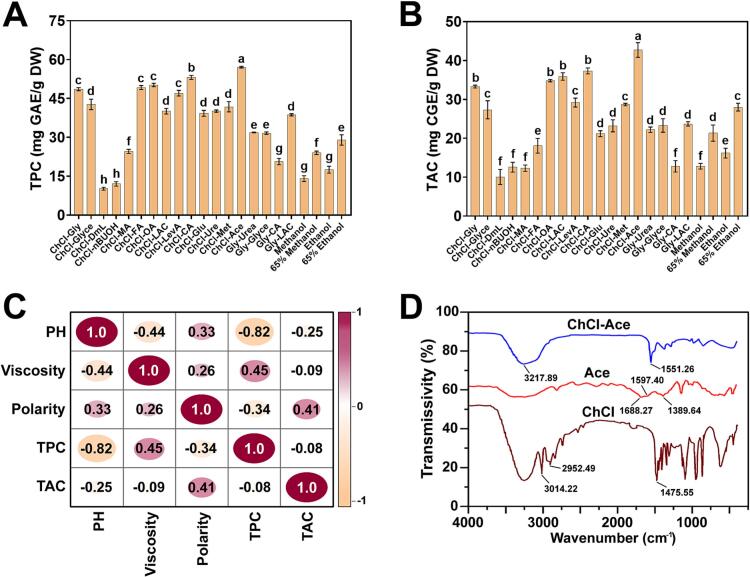
The relationship between the extraction efficiency and DESs is determined by their physico-chemical properties. (A) Total polyphenol content (TPC, measured in mg GAE per g DW) and (B) total anthocyanin content (TAC, measured in mg CGE per g DW). (C) The relationship between the physicochemical properties of DESs (pH, viscosity, and polarity) and TPC and TAC as measured by Pearson correlation coefficients. The correlation coefficient r measures the degree of linear correlation between variables. (D) FT-IR spectra for ChCl–Ace. The results are shown as mean ± SD. Different letters represent statistically significant differences (*p* < 0.05) according to post hoc Tukey’s HSD test.

As alternatives to conventional organic solvents, DESs must demonstrate the capability to efficiently extract biologically active compounds while also offering environmental and economic advantages. The analysis focused on the extraction capabilities of different DESs, and the results are presented in Fig. 1. Quantification of TPC and TAC is presented in Fig. 1A and B, respectively. Among the analyzed groups, group ChCl-Ace demonstrates the highest extraction efficiency of TPC, quantified at 62.66 ± 3.42 mg GAE/g DW, followed by groups ChCl–CA and ChCl–OA, which exhibit extraction efficiencies of 53.42 ± 2.16 mg GAE/g DW and 48.02 ± 2.72 mg GAE/g DW, respectively. ChCl–Ace and ChCl–CA also stand out with the highest TAC values of 43.20 ± 1.22 and 39.42 ± 0.08 mg CGE/g DW, respectively. The effectiveness of ChCl–LAC and ChCl–OA is confirmed by their TAC values of 36.80 ± 1.22 and 34.72 ± 0.84 mg CGE/g DW, respectively. Similar to the TPC findings, DESs such as ChCl–DmL exhibit the lowest TAC, indicating their relative inefficiency for anthocyanin extraction. Extracts with darker hues, such as ChCl–LevA and ChCl–Ace, show higher TAC. The increase in TAC observed with the ChCl-based DESs, particularly when combined with LevA and Ace, is attributed to their effectiveness in breaking down plant cell walls and enhancing anthocyanin solubility [31]. Additionally, studies suggest that ChCl-based DESs are more efficient than traditional solvents for extracting anthocyanins from fruits and vegetables [32]. The ability of DESs to form hydrogen bonds plays a crucial role in stabilizing and protecting anthocyanins, contributing to this phenomenon.

Fig. 1C shows the correlation between the physicochemical properties of the DESs and the TPC and TAC. Specifically, the pH of DES and TPC exhibits a significant negative correlation (r = −0.82, *p* < 0.05), suggesting that acidic conditions may enhance polyphenol extraction by promoting a higher mass transfer rate of phenolic compounds into the solvent [33]. Variations in TPC could be attributed to the presence of different polyphenols such as ellagitannins, catechins, and flavonoids, which react differently under varying pH levels [34]. Furthermore, given that the COF is abundant in ellagitannins and ellagic acid, it is plausible that certain ellagitannins undergo degradation to ellagic acid during extraction. This transformation may result in the increased stability of ellagic acid under varying pH conditions, thereby contributing substantially to the TPC. There was a moderately positive correlation between viscosity and TPC (r = 0.45), whereas the correlation with TAC was weakly negative (r = −0.09). Under these conditions, the weak correlation between polarity and TPC (r = −0.34) and TAC (r = 0.41) indicates that DES's polarity has a limited role in affecting extraction efficiency and antioxidant capacity. In general, pH had a significant impact on TPC, whereas TAC was less influenced by these factors. This advancement is credited to the efficiency of ultrasound in breaking cell walls, boosting mass transfer, and decreasing solvent viscosity, coupled with the remarkable solvation capacity of DESs for polar compounds [21].

In summary, the extraction capacity of a DES is influenced by its composition, fluidity, and polarity and varies depending on the specific compound being extracted and substrate to which it is bound. Consequently, ChCl–Ace was selected as extraction solvent for optimization.

#### 3.1.2 Characterization of ChCl–Ace

The formation of hydrogen bonds between the solvent molecules was verified by analyzing the functional groups of ChCl–Ace and its pure components using FT-IR. As illustrated in Fig. 1D, the spectrum of ChCl exhibits a broad OH stretching vibration peak at 3257 cm^−1^, alongside a distinct CH stretching vibration peak at 3014.22 cm^−1^, as reported by Delgado-Mellado et al. [35]. Additionally, a sharp peak at 1475.55 cm^−1^ is attributed to the bending vibration of CH. The spectrum also reveals a peak at 2952.49 cm^−1^, which is assigned to the stretching vibration of CH_3_, and peaks within the ranges of 2953–2845 cm^−1^ and 1489–1425 cm^−1^, indicative of the presence of alkyl functional groups characterized by CH_2_ [36]. The chemical traits identified in this spectrum agree with the structure of ChCl. Similarly, the spectrum of Ace shows characteristic absorption bands as follows: two medium-intensity peaks at ∼ 3350 cm^−1^ (asymmetric N–H stretch) and ∼3170 cm^−1^ (symmetric N–H stretch), a strong peak at 1688.27 cm^−1^ (C=O stretch, amide I band), and a medium-to-strong peak at 1600–1597.40 cm^−1^ (amide II band, coupling of N–H in-plane bending and C–N stretching). Weak-to-medium absorption bands at 1389.64 cm^−1^ (C–H bending and C–N stretching), and below 1000 cm^−1^ (skeletal vibrations and N–H out-of-plane bending).

In the infrared spectrum of ChCl–Ace, a broad and intense absorption peak appeared at 3217.89 cm^−1^, which was shifted to a lower wavenumber compared with the OH stretching vibration peak at 3256.05 cm^−1^ in the ChCl spectrum. This indicates the formation of intramolecular hydrogen bonds during the synthesis of ChCl–Ace, resulting from the internal associations of the components [37]. The 3600–3200 cm^−1^ band tended to widen and shift to lower wavenumbers from the monomeric compound to ChCl–Ace, suggesting hydrogen bond formation during DES preparation. According to the infrared spectrum of ChCl–Ace, the main functional groups of the monomeric compounds were still present after the formation of the DESs, which is consistent with the conclusion of a previous study [38]. The characteristic functional groups of HBA and HBD also remained undamaged during DES preparation. These spectral alterations unequivocally indicate the formation of hydrogen bonds between the amide group of Ace and the hydroxyl group of ChCl.

### 3.2 Evaluation of ChCI–Ace combined UAE conditions using a single-factor approach

**Fig. 2:**
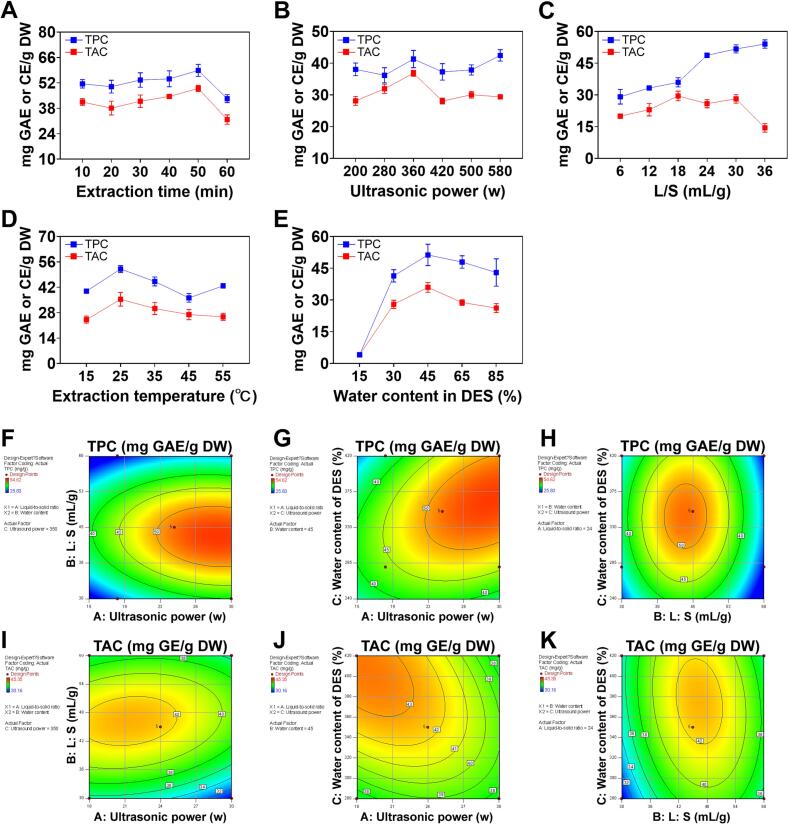
Optimization of extraction conditions using single-factor design and response surface methodology (RSM). Effects of individual variables on TPC and TAC: time of extraction (A), ultrasonic power (B), liquid-to-solid ratio (L/S) (C), temperature of extraction (D), and water content in DESs (E). Surface plots illustrate how ultrasonic power and the liquid-to-solid ratio impact TPC (F-H). Additionally, surface plots show the influence of ultrasonic power and water content in DES on TAC (I-K).

The impact of the liquid-to-solid ratio, temperature, and ultrasonic power on the extraction efficiency was investigated in a single-factor experiment using DESs and UAE, with the results depicted in Fig. 2. Both TPC and TAC showed an initial rise with extraction time, peaking between 10 and 40 min (Fig. 2A). Prolonging the extraction time beyond this range led to reduced efficiency, possibly due to compound breakdown or solvent saturation, as proposed by Rashid R et al. [19]. Similarly, enhancing the UAE power improved the extraction efficiency up to approximately 360 W, beyond which the advantages either plateaued or declined, indicating an optimal UAE power for maximum extraction efficiency (Fig. 2B).

An important aspect of the extraction process is the ratio between the solvent and the solid. Increasing the ratio enhances extraction efficiency due to greater solvent availability for solubilizing compounds. However, ratios exceeding 24:1 to 30:1 (mL/g) did not further improve extraction efficiency significantly (Fig. 2C). Temperature also plays a crucial role, as elevated temperatures enhance extraction yield by improving mass transfer and compound solubility [39]. However, excessively high temperatures led to the breakdown of delicate bioactive compounds, with the optimal extraction temperature found to be around 30 °C (Fig. 2D). Furthermore, the extraction effectiveness was significantly influenced by the water content in the DES, with 45% water content yielding the best results, highlighting the importance of solvent composition in optimizing extraction (Fig. 2E).

### 3.3 Rsm-based optimization of ChCI–Ace–UAE targeting polyphenols

We optimized the extraction conditions by utilizing RSM to explore the interactions of three key parameters: ultrasonic power, liquid-to-solid ratio (L/S), and water content in the DES. As shown in Fig. 2F–2K, these variables significantly influenced the TPC and TAC levels. Notably, significant interactions were noted between ultrasonic power and the sample, as well as between the L/S ratio and water content in the DES, impacting the TPC and TAC levels. Various combinations of ultrasonic power, L/S ratio, and water content were tested to determine the TPC and TAC values (Table S2A). The water content in the DES emerged as a critical factor in the extraction process.

As shown in Table S2A, there was a notable increase in both the TPC and TAC as the water content increased from 30% to 45%, achieving an optimal extraction efficiency of 45%. The solubility of DESs was improved and their viscosity was lowered by the addition of water, which in turn boosted extraction [40]. The reduction in viscosity allowed the polar components to benefit from the varying water proportions. Wang et al. [41] noted that the extraction efficiency was positively affected by the optimal water content of the DESs. In their study, the examination of water ratios ranging from 15% to 45% revealed an increase in TPC as the water content in the DESs increased, which is consistent with our findings. Additional investigations using choline-based DESs have demonstrated that adding more water to the DESs can enhance the efficiency of TPC extraction [31]. Table S2B shows that with increasing water content, the solubilities of TPC and TAC greatly improved, which in turn enhanced the extraction efficiency. A more hydrophilic environment, brought about by the increased water content, might be responsible for this improvement, as it helps dissolve the active compounds. However, when the water content exceeds the optimal level, the structural integrity of the DESs is significantly compromised, resulting in the emergence of a water-like configuration [42]. This structural disruption may account for the diminished extraction efficiency observed for the DESs. Improved solubility and stability of the phenolic compounds were achieved through the interaction of these factors, resulting in higher yields. Based on the RSM analysis, the best conditions for maximizing TPC and TAC extraction involved 350 W ultrasonic power, 24% liquid-to-solid ratio, and 45% water content in the DESs. Under the given conditions, the expected yields were 51.35 mg GAE/g DW for TPC and 42.32 mg CE/g DW for TAC. The actual yields (TPC: 45.22 mg GAE/g DW, TAC: 42.66 mg CE/g DW) obtained through experimentation were in close alignment with the predicted values, validating the RSM model's precision and strength (Table S2A). Furthermore, under conditions optimized by RSM, the findings revealed that TPC and TAC were greatly enhanced compared to the single-factor experimental results. According to Table S2A, the integration of the optimal ultrasonic power, L/S ratio, and water content in DESs leads to superior extraction efficiencies compared to optimizing each factor individually. As shown in Table S2B, the quadratic model's predicted values demonstrated that the quadratic effect of water content (B) significantly influenced the TPC and TAC (*p* < 0.001). Moreover, the combination of ultrasonic power (C) and water content (B), along with the interaction between C and A (L/S ratio), significantly affected the TAC (*p* < 0.05). The R^2^ values of 0.9496 for TPC and 0.9073 for TAC in the predictive models closely matched the observed values.

### 3.4 FTIR analysis of different DESs extracts

**Fig. 3:**
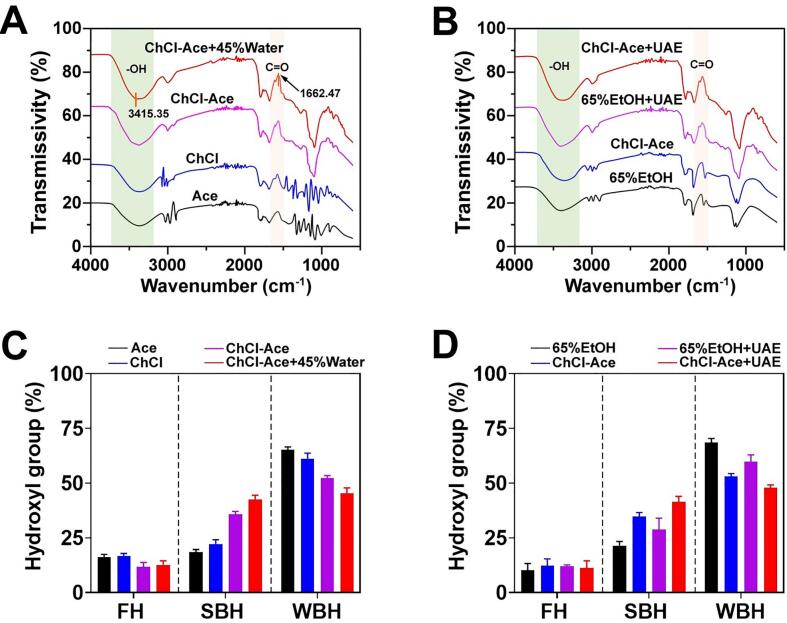
Analyzing the structural characteristics and chemical composition of extracts. (A) FTIR spectra of Ace, ChCl, ChCl–Ace, and ChCl–Ace with 45% water extract. (B) Comparison of FTIR spectra of the COF extracts obtained using various systems including 65%EtOH, ChCl–Ace, 65%EtOH + UAE, and ChCl–Ace + UAE. (C) Percentage of hydroxyl groups for FH, SBH and WBH in FTIR spectra of Ace, ChCl, ChCl–Ace, and ChCl–Ace with 45% water extract. (D) Percentage of hydroxyl groups for FH, SBH and WBH in FTIR spectra of 65%EtOH, ChCl–Ace, 65%EtOH + UAE, and ChCl–Ace + UAE extract.

The FT-IR spectra in Fig. 3 demonstrate the chemical constituents of COF extracts using a range of DESs and UAE methods. The FTIR spectra of the ChCl–Ace solution, containing 45% water, exhibited prominent absorption bands in the O–H stretching (3200–3600 cm^−1^) and C=O stretching (1600–1700 cm^−1^) regions, indicating the presence of phenolic compounds (Fig. 3A). As described in Fig. 3C, the quantitative analysis results showed that, from Ace to ChCl–Ace, the proportion of SBH groups increased significantly from 18.5% to 35.7%, while the proportion of weakly bound hydroxyl groups decreased accordingly. This indicated that when ChCl combined with Ace to form a crystal, the intermolecular hydrogen bond effect was significantly enhanced, thereby constructing a more stable supramolecular network and highlighting the assisted effects of their combination [43]. In the hydrous eutectic (ChCl–Ace + 45% water), the proportion of strongly bound hydroxyl groups was the highest at 42.4%. This indicates that the introduction of water promotes the hydration effect of the hydrogen bond network, forming a broader and stronger hydrogen bond association system and suggesting an enhanced extraction efficiency.

Furthermore, notable variations were observed in the spectra when different extraction solvents and methods were compared (Fig. 3B). Compared to ChCl–Ace alone, the UAE-enhanced ChCl–Ace system exhibited more distinct peaks in the O–H and C=O stretching regions, showcasing the efficacy of ultrasound in enhancing phenolic compound extraction. Similarly, it was observed that the proportion of SBH groups for 65% ethanol with UAE were higher but slightly less than those for ChCl–Ace with UAE (Fig. 3D), suggesting that DESs are more efficient than conventional solvents such as ethanol when exposed to ultrasound [44]. Conversely, the spectra of 65% ethanol and ChCl–Ace without UAE exhibited less proportion of SBH groups, indicating lower extraction efficiencies compared to those achieved with UAE. Ultrasound was proven effective in enhancing extraction efficiency. In summary, the effectiveness of ChCl–Ace in extracting phenolic compounds from COF was significantly improved, particularly when combined with water and UAE, as evidenced by FTIR analysis, supporting the optimization findings.

### 3.5 Analysis of phenolic compound composition

HPLC-ESI-qTOF-MS/MS analysis of COF extracts revealed the presence of eight unique phenolic compounds, including gallic acid, pelargonidin-3-O-glucoside, epicatechin gallate, cyanidin-3-O-glucoside, quercetin, ellagic acid, kaempferol, and delphinidin 3-O-glucoside (Table 2). These compounds were identified by analyzing their retention times, molecular ion peaks, MS/MS fragment ions, molecular weights, and chemical formulas. The accuracy and reliability of the identification process were ensured by maintaining measurement errors within ± 3 ppm. For example, gallic acid (GA) was detected at a retention time of 1.02 min, with a molecular ion at *m*/*z* 171.49 [M + H]^+^ and a distinctive fragment at *m*/*z* 153.01. Similarly, pelargonidin-3-O-glucoside (RT: 2.35 min, *m*/*z* 469.11 [M + H]^+^) and cyanidin-3-glucoside (C3G; RT: 2.69 min, *m*/*z* 450.22 [M + H]^+^) were identified and confirmed through their characteristic MS/MS fragment ions at *m*/*z* 203.05 and *m*/*z* 287.06, respectively. The compound identification procedure relied on reference standards and was supported by existing literature, providing strong evidence for their confirmation.

**Table 2:** Identification and measurement of phenolic compositions in the COF were conducted using the HPLC-ESI-qTOF-MS/MS method. Different letters represent statistically significant differences (p < 0.05) according to post hoc Tukey’s HSD test.

No.	RT (min)	Molecular ion (*m*/*z*)	MS/MS fragment ions (*m*/*z*)	Compound	Formula	MW	Error	References	Contents (μg/g DW)		
									EtOH	ChCl-Ace	ChCl-Ace + UAE
1	1.02	171.49 [M + H]^+^	171.49, 153.01, 196.28	Gallic acid	C_7_H_6_O_5_	170	0.5	Standard, MS/MS	175.26 ± 5.87^a^	1002.65 ± 19.69^b^	1315.14 ± 18.51^c^
2	2.35	469.11[M + H]^+^	469.11, 176.09, 203.05	Pelargonidin-3-O-glucoside	C_21_H_21_ClO_10_	468	0.1	Standard, MS/MS	4.56 ± 0.24^a^	8.64 ± 0.66^b^	25.27 ± 3.28^c^
3	2.69	450.22[M + H]^+^	450.22, 367.01, 287.06, 109.02	Cyanidin-3-O-glucoside	C_21_H_21_ClO_11_	449	0.3	Standard, MS/MS	82.47 ± 1.58^a^	438.28 ± 9.36^b^	946.83 ± 8.22^c^
4	3.21	441.13[M-H]^-^	441.13, 333. 25, 169. 09, 125.06	Epicatechin gallate	C_22_H_18_O_10_	441	0.2	Standard, MS/MS	59.01 ± 1.36^a^	327.07 ± 7.24^b^	809.62 ± 6.65^c^
5	5.39	303.37[M + H]^+^	303.37, 285.29, 260.07	Quercetin	C_15_H_10_O_7_	302	−1.2	Standard, MS/MS	0.55 ± 0.03^a^	1.44 ± 0.07^b^	3.37 ± 0.43^c^
6	5.97	466.14[M + H]^+^	466.14, 303. 21, 127.12	Delphinidin-3-O-glucoside	C_21_H_21_O_12_	465	−0.7	Standard, MS/MS	0.49 ± 0.02^a^	1.15 ± 0.04^b^	2.93 ± 0.38^c^
7	7.74	285.23[M-H]^-^	285.23, 267. 03, 257. 13, 229. 41	Kaempferol	C_15_H_10_O_6_	286	0.4	Standard, MS/MS	7.23 ± 0.46^a^	12.06 ± 1.77^b^	20.05 ± 2.29^c^
8	10.66	303.12[M + H]^+^	303.12, 193.32, 153.10	Ellagic acid	C_14_H_6_O_8_	302	−0.5	Standard, MS/MS	13.44 ± 0.33^a^	244.17 ± 5.66^b^	451.56 ± 4.72^c^

Quantitative analysis revealed that the extraction solvent used caused significant variations in the phenolic compound concentrations. The ChCl–Ace–UAE system exhibited notably higher concentrations of most phenolic compounds compared to both ChCl–Ace and EtOH extraction methods. Specifically, the ChCl–Ace–UAE extract contained GA at a concentration of 1315.14 ± 18.51 μg/g, surpassing levels in ChCl–Ace (1002.65 ± 19.69 μg/g) and EtOH (82.47 ± 1.58 μg/g). Similarly, the epicatechin gallate content was significantly higher in the ChCl–Ace–UAE extract (809.62 ± 6.65 μg/g) than those in ChCl–Ace (327.07 ± 7.24 μg/g) and EtOH (59.01 ± 1.36 μg/g). A similar trend was observed for ellagic acid, with the ChCl–Ace–UAE system yielding the highest concentration (451.56 ± 4.72 μg/g), surpassing ChCl-Ace (244.17 ± 5.66 μg/g) and EtOH (13.44 ± 0.33 μg/g). The improved extraction efficiency of the ChCl–Ace–UAE system stemmed from the synergy between ultrasonic cavitation and the unique physicochemical properties of the ChCl–Ace eutectic solvent. This interaction results in an overall enhancement of the solubility and mass transfer of phenolic compounds [45], [46].

**Fig. 4:**
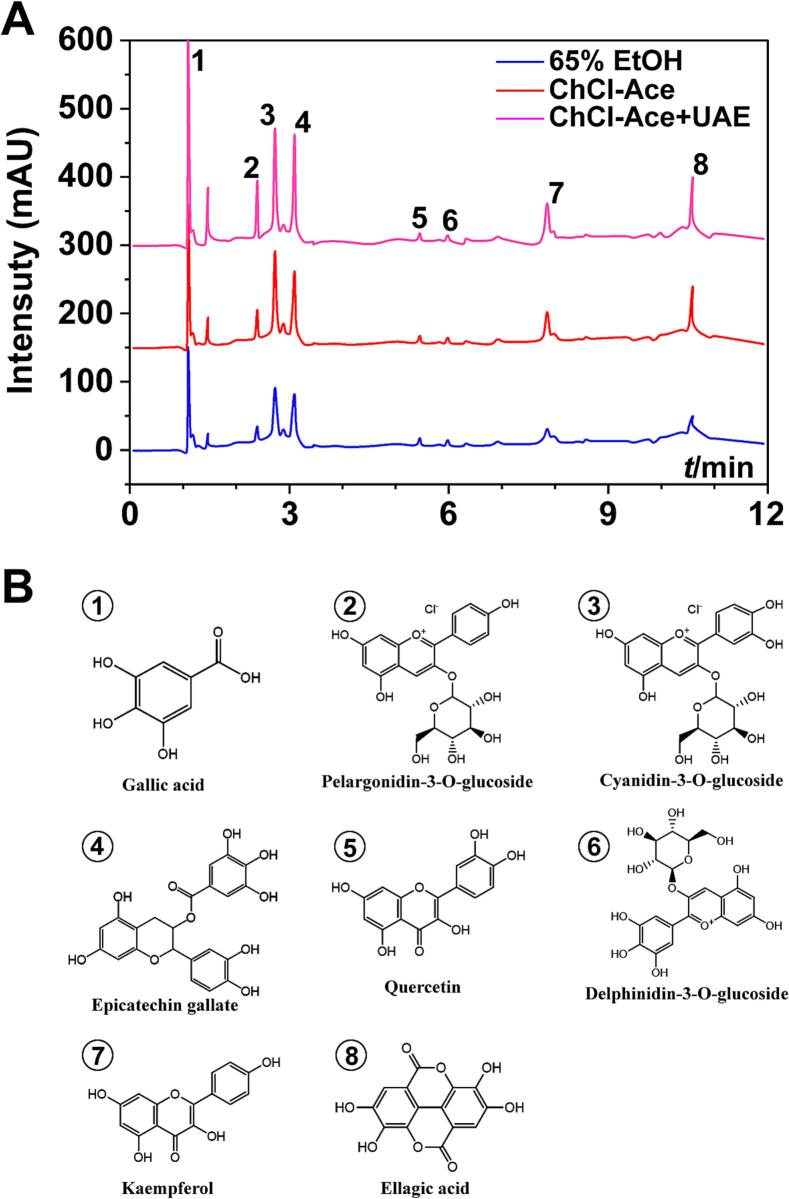
COF extract HPLC chromatograms obtained with 65%ethanol, ChCl–Ace, and ChCl–Ace + UAE (A). The chemical molecular structure formula of polyphenolic compunds identified in COF(B).

The phenolic compound profile initially recognized by HPLC-ESI-qTOF-MS/MS was later confirmed using conventional HPLC methods (Fig. 4). According to the HPLC chromatogram, eight phenolic compounds were present with retention times that corresponded closely to the mass spectrometry findings. Significantly, the ChCl–Ace–UAE extract exhibited the most pronounced peak intensities for essential phenolic compounds, such as GA (peak 1), C3G (peak 3), epicatechin gallate (peak 4), and ellagic acid (peak 8), exceeding those observed in both the non-ultrasonicated ChCl–Ace and ethanol extracts. Conversely, the ethanolic extract exhibited significantly diminished peak intensities, indicating a reduced capacity to extract these phenolic compounds. Without ultrasound, the ChCl-Ace system showed moderate extraction performance, highlighting the benefits of ultrasonication in enhancing extraction efficiency.

### 3.6 SEM analysis

**Fig. 5:**
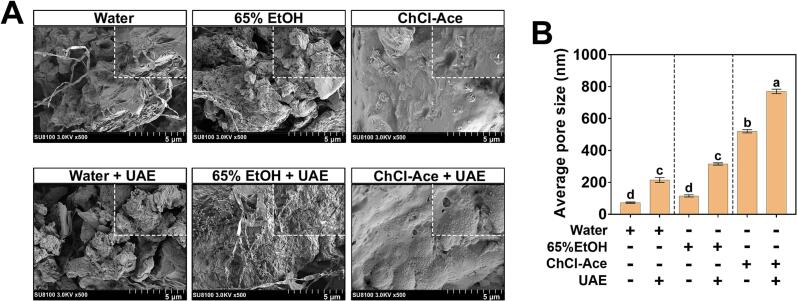
Images from SEM showing COF after undergoing different treatments: water, 65%ethanol, ChCl–Ace, water + UAE, 65%ethanol + UAE, and ChCl–Ace + UAE extract (A). Statistical analysis for average pore size of COF extract (B). The results are shown as mean ± SD. Different letters represent statistically significant differences (*p* < 0.05) according to post hoc Tukey’s HSD test.

The SEM images of the COF extracts obtained using different solvents, such as DES, 65% ethanol, and water under UAE, revealed distinct effects on cell morphology and extraction efficiency (Fig. 5A). The utilization of ChCl–Ace in UAE led to the most significant structural changes, characterized by the development of visible pores and fissures in the cell walls. In detail, the statistical results of COF extracts showed that the ChCl–Ace–UAE group had a smaller average pore size (770 ± 14.32 nm) than those of the water (73 ± 6.16 nm), water–UAE (215 ± 17.55 nm) and ChCl–Ace (520 ± 22.38 nm) groups (Fig. 5B). This enhanced porosity facilitated better solvent infiltration, resulting in a higher yield of extracted bioactive compounds, including phenolics and flavonoids [47]. A similar phenomenon was observed in a study by Wang et al. [41].

When combined with UAE, 65% ethanol exhibited enhanced performance, causing moderate cell wall disruption and increased extraction efficiency compared with ethanol without sonication (Fig. 5). However, its efficacy was lower than that of ChCl–Ace, likely due to ethanol’s limited capacity to degrade cellular structures to release the active ingredients. In contrast, utilizing UAE with water led to minimal cellular disruption, resulting in minor changes in COF cells. The extraction efficiency was poorest when water served as the solvent, indicating its limited ability to extract bioactive compounds. In the absence of UAE, all solvents caused minimal cell wall disruption, leading to decreased extraction efficiencies. This highlights the crucial role of UAE in improving extraction performance by facilitating the breakdown of cellular structures and enhancing solvent infiltration. In conclusion, employing ChCl–Ace with UAE yielded the most efficient extraction, attributed to its superior ability to disrupt cell walls and the assisted interaction between the solvent and extraction technique. This synergy optimizes the release of target compounds, establishing DES as the most effective extraction medium.

### 3.7 MD simulations

**Fig. 6:**
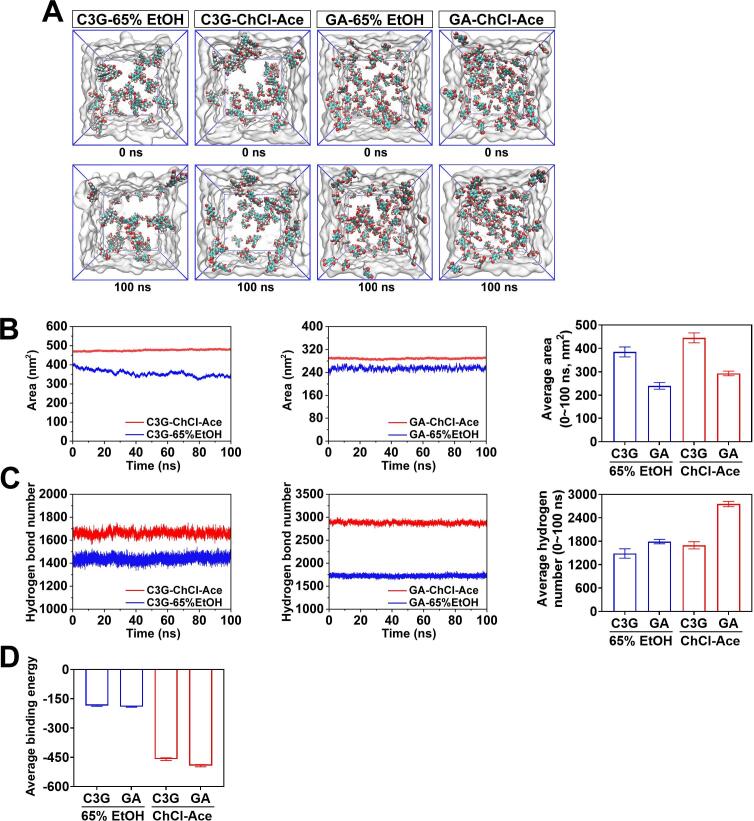
Exploring the extraction mechanism through MD simulations. (A) Snapshots of C3G or GA molecules in ChCl–Ace and EtOH solutions. (B) Solvent-exposed surface area (SASA) along with its average. (C) the total hydrogen bonds and their corresponding average amounts. (D) Variation in the average binding energy in different DESs over a timespan of 0–100 ns.

At 0 and 100 ns, snapshots of the molecular system (Fig. 6A) depicted the stability and structural characteristics of C3G and GA in 65% ethanol and ChCl–Ace. The simulation highlighted notable disparities in the packing and interactions of the molecules within the solvents [26]. Molecular configurations in the ChCl–Ace system appeared more condensed at 100 ns, demonstrating enhanced organization compared to 65% ethanol. The distinct packing density in ChCl–Ace was attributed to specific interactions between ChCl and Ace, which facilitated closer proximity between the molecules, thereby improving the efficiency of the extraction process.

Notably, the solvent effects became apparent through temporal changes in the area of the system (Fig. 6B). The average area in the C3G system is considerably smaller in ChCl–Ace (483.62 nm^2^) than in 65% ethanol (397.47 nm^2^), pointing to more solid molecular interactions and a tighter arrangement within the eutectic solvent [27]. A smaller area implies a more beneficial solvation environment for ChCl–Ace, with the solvent molecules adhering more strongly to C3G, thereby stabilizing its structure and improving its solubility. Similarly, the GA system demonstrates a decreased area in ChCl–Ace (203.94 nm^2^) compared to 65% ethanol (233.92 nm^2^), highlighting the significance of ChCl–Ace in facilitating molecular interaction and aggregation.

The establishment of hydrogen bonds serves as a key indicator of the molecular interactions in these systems (Fig. 6C) [48]. With an average of 1754.32 hydrogen bonds, the C3G–ChCl–Ace system surpasses the C3G–65% EtOH system, which has 1483.26, highlighting the greater potential of the eutectic solvent for hydrogen bonding. In the ChCl–Ace system, the increased number of hydrogen bonds implies that the ChCl and Ace components formed a hydrogen-rich setting, facilitating more frequent and robust hydrogen bond interactions with C3G. Similarly, the GA-ChCl-Ace system produced the largest number of hydrogen bonds (2795.24), outperforming the GA–65% EtOH system (1851.45). This indicates that ChCl–Ace creates conditions that strengthen and increase the frequency of hydrogen bonding interactions [47]. Electrostatic forces between the choline chloride cation and phenolic hydroxyl groups may be part of the interactions between GA and the solvent, aiding in the stabilization of the GA molecule in the solution.

A deeper understanding of the interactions between the solutes and solvents at the molecular level was provided by the binding-energy results (Fig. 6D). The binding energy of the C3G–ChCl–Ace system is considerably lower at −459.78 kJ/mol than that of the C3G-65% EtOH system at −185.05 kJ/mol, indicating that the eutectic system has stronger interactions between the solvent and solute. In ChCl–Ace, a more energetically favorable interaction with the phenolic compound is suggested by the lower binding energy, implying that the solvent mixture provides better stabilization of the solute molecules and diminishes the total energy of the system. This difference in binding energy directly translates into a difference in extraction efficiency. The key link is the change in the number and stability of hydrogen bonds. The more negative the binding energy of a system, the more hydrogen bonds that are formed between the polyphenol and solvent, and the higher the hydrogen bond occupancy [46]. In the ChCl–Ace system, the phenolic hydroxyl groups of the polyphenol act as hydrogen bond donors and can form a large number of stable hydrogen bonds with chloride ions (strong hydrogen bond acceptors) as well as the carbonyl group (C=O) and amino group (–NH_2_) of acetamide in DES. Simultaneously, the hydroxyl groups of the choline cation can form secondary hydrogen bonds with the polyphenol, and the multiple hydrogen bonds further strengthen the binding stability between the polyphenol and solvent. In the 65% ethanol system, only a small number of hydrogen bonds were formed between the hydroxyl groups of the ethanol molecules and the polyphenol, and the hydrogen bond lifetime was short and prone to breakage. These explanations confirm the significant advantage of the DES extraction system over the CSE method for the extraction of polyphenols. Similarly, in the context of GA systems, we found that ChCl–Ace has a binding energy of −493.21 kJ/mol, which is significantly lower than that observed in 65% EtOH (−191.02 kJ/mol). This finding supports the hypothesis that ChCl–Ace strengthens the binding affinity between GA and solvent molecules, culminating in a more stable and tightly arranged molecular structure, and also presented better extraction efficiency. These findings support earlier research confirming the superior molecular affinity and stability of DES [47].

One point that deserves attention in MD is that we used the Amber ff99SB force field (a well-validated force field mainly for proteins) and the ACPYPE web server to generate parameters for chloroform, acetic acid, and phenolic glycosides [22], [25]. Amber ff99SB is primarily parameterized for proteins, and its general parameters may not be fully optimized for the unique functional groups in ChCl (quaternary ammonium), acetone, and phenolic glycosides (aromatic rings linked to glycosyl moieties), which could introduce minor deviations in the description of intramolecular vibrations and intermolecular van der Waals interactions [26]. Therefore, we acknowledge that ff99SB is not specifically optimized for the unique functional groups of these non-standard molecules, which may affect the parameter optimality. In addition, the use of general ACPYPE parameters lacks compound-specific refinements for the target phenolic glycosides, which may affect the quantitative precision of the interaction energies and dynamic behavior compared to the force fields fitted specifically to these molecules [46]. Therefore, considering the above factors, further research to optimize the parameters to enhance the accuracy of the simulation and overcome the limitations caused by the Amber ff99SB force field and ACPYPE needs to be conducted in the future.

### 3.8 *In vitro* comparison of antioxidant activity

**Fig. 7:**
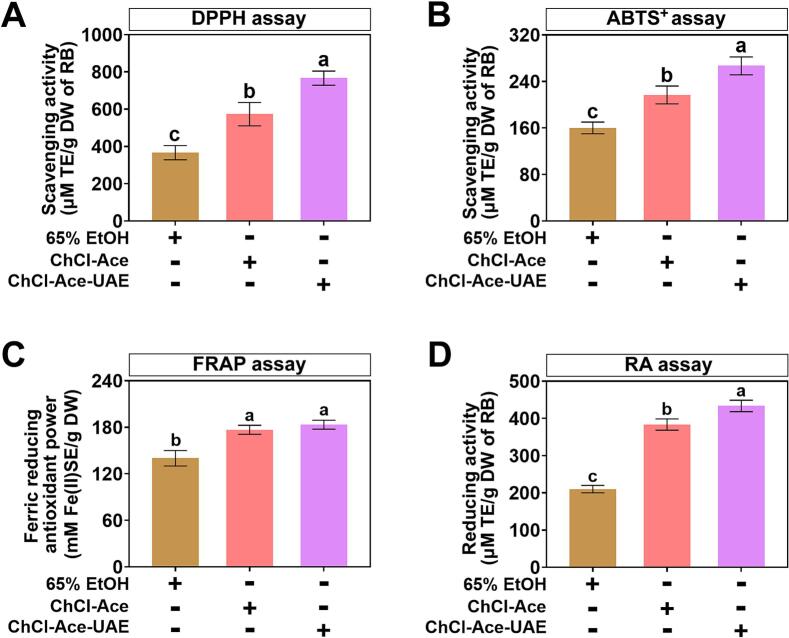
DPPH radical elimination activity (A), ABTS + radical elimination activity (B), ferric reducing antioxidant strength (FRAP) (C), and reduction potential (RA) (D). The results are shown as mean ± SD. Different letters represent statistically significant differences (*p* < 0.05) according to post hoc Tukey’s HSD test.

Fig. 7A–7D evaluated the antioxidant properties of the COF extracts generated through various extraction methods. Among these, extracts produced using ChCl–Ace–UAE exhibited the highest antioxidant activity in all experiments, while the 65% ethanol extract exhibited the lowest antioxidant activity throughout all experiments. Specifically, DPPH and ABTS^+^ measurements indicated significant enhancements in free radical scavenging capacity (Fig. 7A–7B). Similarly, the ChCl–Ace–UAE extract demonstrated superior reducing capabilities in the FRAP and reducing power assays, with statistically significant results (*p* < 0.01) (Fig. 7C–7D). Extracts obtained using ChCl–Ace without ultrasound also showed notable antioxidant activity, albeit at relatively lower levels compared to those obtained with ultrasound assistance [49]. Moreover, all assays (DPPH, ABTS^+^, FRAP, and reducing power) revealed that the extracts derived using DES exhibited significantly higher antioxidant activity (*p* < 0.01) than those obtained using 65% ethanol, highlighting the effectiveness of DES even without ultrasound.

In all assays, the 65% EtOH extract exhibited the lowest antioxidant activity, significantly lower than values obtained using other methods. These results highlight the superior efficiency of DES in extracting potent antioxidant compounds from COF, particularly when combined with ultrasound. The ChCl-Ace-UAE extract showed enhanced antioxidant properties, likely due to assisted interaction between DES and ultrasound. Ultrasound-aided mass transfer and cellular disruption, increasing the yield and concentration of bioactive constituents [50]. Collectively, these findings indicate that ultrasound-assisted DES extraction is an effective method for obtaining superior COF extracts with improved antioxidant capabilities.

### 3.9 Effects of various extracts on H_2_O_2_-induced oxidative damage in RAW264.7 cells

**Fig. 8:**
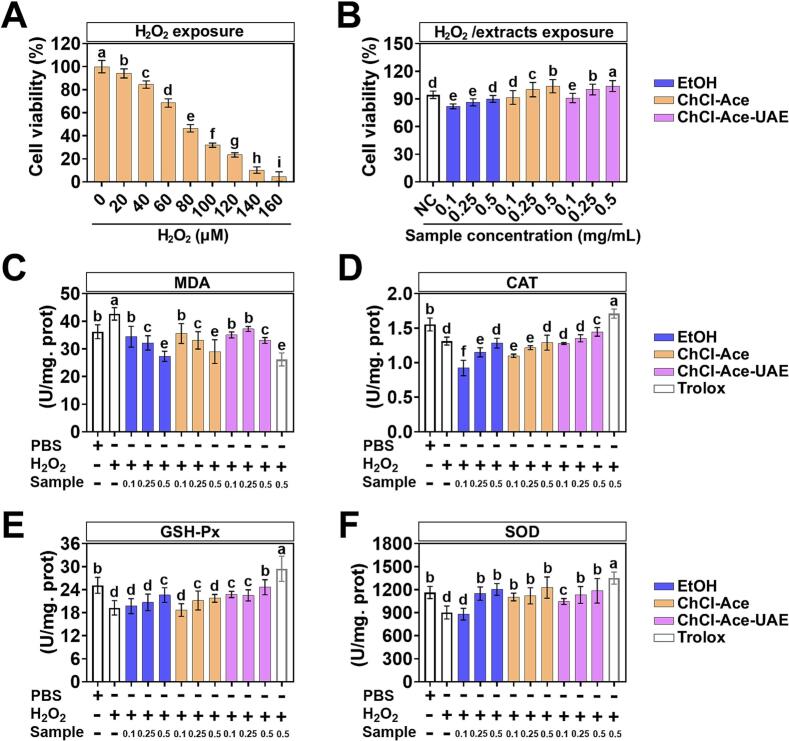
(A-B) RAW264.7 cells underwent exposure to different extracts and a range of H_2_O_2_ concentrations for 24 h. Effect of COF extracts using different DESs on MDA content (C), CAT (D), GSH-Px (E), and SOD (F) activity in RAW264.7 cells. The results are shown as mean ± SD. Different letters represent statistically significant differences (*p* < 0.05) according to post hoc Tukey’s HSD test.

The impact of various extracts on oxidative damage induced by H_2_O_2_ in RAW264.7 cells was examined in Fig. 8. A decrease in RAW264.7 cell viability was noted following exposure to H_2_O_2_ through cell viability assays, thereby validating the successful induction of oxidative stress (Fig. 8A). Among the tested treatments, ChCl–Ace–UAE at all concentrations demonstrated superior cytoprotective efficacy compared to both the 65% ethanol and ChCl–Ace extracts (Fig. 8B). UAE was observed to modify the surface structure of the samples, as revealed by the SEM results, facilitating the release of phenolic compounds.

In this study, an H_2_O_2_-induced oxidative stress model in RAW264.7 cells was utilized to further assess the impact of the extracts on essential cellular antioxidant markers, including MDA levels and the enzymatic activities of CAT, GSH-Px, and SOD. Overall, a significantly higher antioxidant activity was observed in the positive control Trolox compared to the extract treatments (*p* < 0.05) (Fig. 8C–8F). Importantly, MDA concentrations were notably reduced by all extracts in cells exposed to H_2_O_2_. The ChCl–Ace–UAE extract exhibited the most substantial decrease, emphasizing its potent inhibition of lipid peroxidation (Fig. 8C). Furthermore, measurements were taken for the activities of the antioxidant enzymes CAT, GSH-Px, and SOD (Fig. 8D–8F). A significant enhancement in enzyme activity was observed following the ChCl–Ace–UAE treatment compared to the oxidative stress model group (*p* < 0.05). Notably, cells exposed to 0.5 mg/mL ChCl–Ace–UAE showed significantly elevated CAT activity, facilitating the breakdown of H_2_O_2_ into water and oxygen, thereby alleviating oxidative stress [51]. Following the ChCl–Ace–UAE treatment, the activities of GSH-Px and SOD increased significantly, indicating that the enriched antioxidant constituents within this extract potentiated enzymatic antioxidant defenses and contributed to the maintenance of cellular redox homeostasis [46].

Notably, an interaction exists between oxidative stress and the inflammatory response [30]. On one hand, inflammatory cells produce ROS that participate in oxidative stress; on the other hand, ROS can lead to increased expression of inflammatory cytokines [33]. Besides, ROS can be used as the second messenger downstream of some special ligands (TGF-β, EGF-2, and PDGF) to participate in the regulation of intracellular inflammatory signal transduction pathway, and they also regulate the activity of some inflammatory transcription factors (such as NF-κB) [16]. Therefore, we inferred that COF extracts may also have great anti-inflammatory potential. Considering that the inflammation-related bioactivity of plant extracts, especially polyphenols, has become the current interest, if conditions permit, relevant anti-inflammatory data should be provided to further confirm the anti-inflammatory potential of Schisandra chinensis polyphenols.

Collectively, our results indicate that the ChCl–Ace–UAE extraction method is an effective approach for optimizing the recovery of antioxidant compounds from COF, with promising potential applications in the food and nutraceutical sectors.

### 3.10 Antibacterial activity

**Fig. 9:**
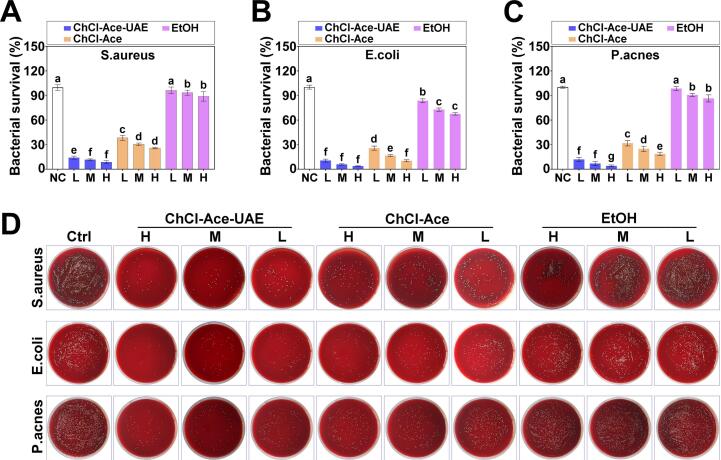
Effect of COF extracts using different DESs on *S. aureus* (A), *E. coli* (B), *and P. acnes* (C). (D) A comparative chart of the antimicrobial activity of COF extracts *via* blood agar culture. The results are shown as mean ± SD. Different letters represent statistically significant differences (*p* < 0.05) according to post hoc Tukey’s HSD test.

This study investigated the antibacterial properties of various extracts using the plate counting method (Fig. 9). The experimental results revealed significant antibacterial activity against *S. aureus*, *E. coli*, and *P. acnes* for all extracts compared to the blank, with effectiveness varying based on concentration. Particularly, the ChCl–Ace–UAE extract exhibited superior antibacterial effects compared to ChCl–Ace and EtOH. At a low loading concentration of the ChCl–Ace–UAE extract (0.1 mg/mL), inhibition rates for *S. aureus*, *E. coli*, and *P. acnes* were 83.26 ± 2.12%, 86.19 ± 1.71%, and 89.05 ± 2.44%, respectively. Notably, although both *S. aureus* and *P. acnes* are Gram-positive bacteria, the inhibition rate of *P. acnes* was considerably lower. This suggests that antibacterial activity may be influenced by factors beyond bacterial classification, potentially involving unique characteristics of each species, such as cell wall composition or metabolic traits [30]. Upon increasing the concentration to 0.5, the inhibition rates significantly increased to 91.25 ± 0.15% for *Staphylococcus aureus*, 95.48 ± 0.17% for *Escherichia coli*, and 93.72 ± 0.36% for *Propionibacterium acnes*, indicating a strong positive correlation between ChCl-Ace-UAE extract and antibacterial efficacy.

An in-depth analysis, including literature reviews, suggested that the enhanced inhibition of gram-positive bacteria such as *S. aureus* by the ChCl–Ace–UAE extract could be linked to structural variations in their cell walls [30], [52]. Gram-negative bacteria have multiple barriers composed of a lipopolysaccharide outer membrane, pore proteins, and an active efflux system that significantly reduce the penetration efficiency and intracellular accumulation of polyphenol active components and DESs. In contrast, Gram-positive bacteria lack outer membrane protection and have relatively higher cell wall permeability. Polyphenols can rapidly penetrate the cell wall with the assistance of DESs and target the destruction of the cell membrane structure, a mechanism that aligns with the findings of previous studies. The minimal inhibitory effect observed on *Propionibacterium acnes*, despite its classification as a Gram-positive bacterium [52]. Based on the characteristics of the DES-assisted extracts, the specific explanations may be related to specific modification of the cell membrane structure and a unique metabolic regulation mechanism. As a Gram-positive bacteria, although *Propionibacterium acnes* has no outer membrane, the composition of cell membrane lipids may be altered (such as increasing the proportion of hydrophobic lipids) to reduce the binding and penetration ability of polyphenols [33]. In addition, *Propionibacterium acnes* can resist the oxidative stress and metabolic inhibition induced by polyphenols through its own metabolic regulation (such as activating stress metabolic pathways and enhancing ATP synthesis efficiency), thereby enhancing its tolerance to polyphenol DES extracts [53]. Collectively, these findings validate the essential function of the ChCl–Ace–UAE extract and underscore its selective inhibition of Gram-positive pathogens at reduced levels, along with its broad-spectrum antimicrobial capabilities at increased concentrations [54]. This study provides crucial experimental findings for the development of targeted anti-infection agents.

## 4 Conclusions

The current study showed that ChCl–Ace (1: 2) optimized the conditions for ultrasound-assisted extraction, achieving optimal results with an ultrasonic power of 350 W, a solid:liquid ratio of 24: 1 mL/g, and a water addition of 45% at 45 °C. Eight compounds identified in the COF pulp were gallic acid, pelargonidin-3-O-glucoside, cyanidin-3-O-glucoside, epicatechin gallate, quercetin, delphinidin-3-O-glucoside, kaempferol, and ellagic acid. The results of SEM analysis indicated that DESs disrupted plant cell walls, facilitating the dissolution of phenolic compounds with ultrasound assistance. The interactions between the solute and solvent were notably enhanced by ChCl–Ace, leading to a reduction in the system area, increased hydrogen bonding, and decreased binding energy. *In vitro* assays for antioxidant and antibacterial activities revealed that the ChCl–Ace–UAE extract exhibited significantly higher antioxidant activity compared to extracts obtained through alternative methodologies. In summary, these results highlight the benefits of utilizing assisted ultrasonic DESs to enhance the extraction of bioactive phenolic compounds from COF in line with sustainable and environmentally friendly extraction practices.

Another potential limitation of this study is the non-recovery of eutectic components from the extracts post-extraction. Although these components are non-toxic, their removal depends on the intended application of the extracts. Various methods for separating the extracted phenolic compounds from the NADES eutectic components have been described in the literature, such as recrystallization, adsorption chromatography, and antisolvent applications. Our research focused on developing a new and optimized extraction technique for COF phenolics; therefore, we did not proceed with a recovery study. Therefore, future research should focus on evaluating the recyclability of NADES, expanding the production methods, and employing *in vivo* validation to translate laboratory findings into clinical effectiveness.

## 5 Author Statement

Chen P, WH Xiao, RX Li, BH Zhou, FC Chen and RH Zhang conceptualized and designed the study, analyzed and interpreted data. Chen P wrote the manuscript. Chen P and WH Xiao designed the figures. Chen P, WH Xiao, RX Li, BH Zhou, FC Chen and RH Zhang acquired the data. Chen P and RH Zhang reviewed the manuscript. All authors read and approved the final manuscript. All authors have participated sufficiently in the work and agreed to be accountable for all aspects of the work.

## CRediT authorship contribution statement

**Peng Chen:** Project administration, Visualization, Validation, Supervision, Software. **Wenhao Xiao:** Project administration, Methodology, Investigation. **Ruixiang Li:** Methodology, Investigation. **Benhong Zhou:** Visualization, Validation, Supervision, Software. **Fuchao Chen:** Validation, Supervision, Software, Resources. **Ruhong Zhang:** Visualization, Validation, Supervision, Software.

## Declaration of competing interest

The authors declare that they have no known competing financial interests or personal relationships that could have appeared to influence the work reported in this paper.

## Appendix A Supplementary data

Supplementary Data 1 Physical-chemical properties of DES and RSM design and predicted results in this study.
