# Supplementary material for: Ultrasound-assisted deep eutectic solvent extraction of polyphenols from Cornus officinalis: Optimization, mechanisms, and bioactivity
Source: Ultrason Sonochem. 2026 Jun 1;130:107909. doi: 10.1016/j.ultsonch.2026.107909 (PMC13265695; doi:10.1016/j.ultsonch.2026.107909)
Supplement: Supplementary Data 1 — Physical-chemical properties of DES and RSM design and predicted results in this study. [file mmc1.docx]

**Table S1** Lists and physical-chemical properties of deep eutectic solvent (DES) prepared in this study.

| No. | Component A | Component B | Abbreviations | Molar ratio  (mol/mol) | pH | Viscosity  (mPa·s, 30% Water) | Polarity | Heating duration (min, at 80 °C) |
| --- | --- | --- | --- | --- | --- | --- | --- | --- |
| DEs-1 | Choline chloride | Glycol | ChCl-Gly | 1:2 | 1.44 | 568.63 | 48.24 | 30 |
| DEs-2 | Choline chloride | Glycerol | ChCl-Glyce | 1:2 | 1.85 | 572.39 | 42.37 | 60 |
| DEs-3 | Choline chloride | D-mannitol | ChCl-DmL | 1:2 | 2.99 | 334.55 | 28.36 | 90 |
| DEs-4 | Choline chloride | N-butanol | ChCl-nBUOH | 1:1 | 3.43 | 347.68 | 36.89 | 120 |
| DEs-5 | Choline chloride | Malonate | ChCl-MA | 1:1 | 4.62 | 574.63 | 34.53 | 90 |
| DEs-6 | Choline chloride | Formic acid | ChCl-FA | 1:2 | 3.29 | 585.74 | 49.22 | 45 |
| DEs-7 | Choline chloride | Oxalic acid | ChCl-OA | 1:2 | 2.77 | 612.44 | 50.81 | 90 |
| DEs-8 | Choline chloride | Lactic acid | ChCl-LAC | 2:1 | 4.12 | 591.65 | 40.46 | 45 |
| DEs-9 | Choline chloride | Levulinic acid | ChCl-LevA | 1:4 | 5.35 | 574.67 | 47.38 | 30 |
| DEs-10 | Choline chloride | Citric acid | ChCl-CA | 1:2 | 6.46 | 538.25 | 53.54 | 30 |
| DEs-11 | Choline chloride | Glucose | ChCl-Glu | 1:1 | 3.23 | 584.31 | 38.73 | 50 |
| DEs-12 | Choline chloride | Urea | ChCl-Ure | 2:1 | 3.63 | 577.94 | 40.36 | 30 |
| DEs-13 | Choline chloride | Methylurea | ChCl-Met | 1:2 | 4.27 | 569.16 | 44.71 | 60 |
| DEs-14 | Choline chloride | Acetamide | ChCl-Ace | 1:2 | 4.46 | 642.64 | 57.25 | 45 |
| DEs-15 | Glycine | Urea | Gly-Urea | 1:1 | 2.68 | 498.87 | 38.44 | 60 |
| DEs-16 | Glycine | Glycerol | Gly-Glyce | 1:1 | 3.52 | 506.36 | 38.28 | 55 |
| DEs-17 | Glycine | Citric acid | Gly-CA | 1:1 | 4.34 | 494.82 | 32.32 | 40 |
| DEs-18 | Glycine | Lactic acid | Gly-LAC | 1:1 | 2.66 | 530.53 | 39.59 | 120 |
