# Supplementary material for: Ultrasound-assisted deep eutectic solvent extraction of polyphenols from Cornus officinalis: Optimization, mechanisms, and bioactivity
Source: Ultrason Sonochem. 2026 Jun 1;130:107909. doi: 10.1016/j.ultsonch.2026.107909 (PMC13265695; doi:10.1016/j.ultsonch.2026.107909)
Supplement: Supplementary Data 2 [file mmc2.docx]

**Table S2A**

RSM design and predicted results for the optimization of TPC and TAC

| Factors |  |  | Areb. | Unit | Actual levels | | |  | | |
| --- | --- | --- | --- | --- | --- | --- | --- | --- | --- | --- |
|  |  |  |  |  |  | -1 | 0 | | +1 | |
| Liquid to solid ratio (L/S) | | | A | mL/g |  | 18 | 24 | | 30 | |
| Water content in DES | | | B | % |  | 30 | 45 | | 60 | |
| Ultrasonic power | | | C | W |  | 280 | 350 | | 420 | |
| Run. | A | B | C |  |  | TPC (mg GAE/g DW) | | TAC (mg CE/g DW) | | |
|  |  |  |  |  |  | Exp. | Pred. | Exp. | | Pred. |
| 1 | 24 | 30 | 280 |  |  | 32.54 | 33.99 | 31.59 | | 29.82 |
| 2 | 24 | 60 | 420 |  |  | 26.78 | 25.33 | 34.17 | | 35.94 |
| 3 | 30 | 60 | 350 |  |  | 27.47 | 30.33 | 35.98 | | 35.48 |
| 4 | 18 | 30 | 350 |  |  | 32.43 | 29.56 | 34.92 | | 35.43 |
| 5 | 24 | 45 | 350 |  |  | 45.22 | 51.35 | 42.66 | | 42.32 |
| 6 | 18 | 60 | 350 |  |  | 25.83 | 24.62 | 36.27 | | 36.33 |
| 7 | 30 | 45 | 280 |  |  | 48.37 | 45.71 | 35.62 | | 37.45 |
| 8 | 18 | 45 | 420 |  |  | 33.62 | 36.28 | 40.35 | | 43.15 |
| 9 | 30 | 45 | 420 |  |  | 41.72 | 40.30 | 38.28 | | 37.02 |
| 10 | 24 | 60 | 280 |  |  | 25.94 | 25.73 | 36.67 | | 35.34 |
| 11 | 30 | 30 | 350 |  |  | 38.68 | 39.89 | 30.16 | | 30.10 |
| 12 | 24 | 30 | 420 |  |  | 31.37 | 31.57 | 33.84 | | 35.17 |
| 13 | 24 | 45 | 350 |  |  | 42.06 | 50.33 | 41.17 | | 42.32 |
| 14 | 24 | 45 | 350 |  |  | 40.21 | 41.54 | 30.28 | | 32.32 |
| 15 | 24 | 45 | 350 |  |  | 44.62 | 41.32 | 32.53 | | 30.11 |
| 16 | 18 | 45 | 280 |  |  | 42.28 | 43.69 | 35.87 | | 37.14 |
| 17 | 24 | 45 | 350 |  |  | 41.65 | 44.36 | 43.71 | | 32.25 |
| Fitting formula | | | | | | | | | | |
| TPC = -213.95 + 2.34A + 6.91B + 0.46C - 0.013AB + 0.0071AC + 0.00048BC - 0.075A^2^ - 0.078B^2^ - 0.00095C^2^  TAC = -133.01 + 2.79A + 2.74B + 0.44C + 0.012AB - 0.0041AC - 0.0011BC - 0.045A^2^ - 0.028B^2^ - 0.00039C^2^ | | | | | | | | | | |
