# Supplementary material for: Ultrasound-assisted deep eutectic solvent extraction of polyphenols from Cornus officinalis: Optimization, mechanisms, and bioactivity
Source: Ultrason Sonochem. 2026 Jun 1;130:107909. doi: 10.1016/j.ultsonch.2026.107909 (PMC13265695; doi:10.1016/j.ultsonch.2026.107909)
Supplement: Supplementary Data 3 [file mmc3.docx]

**Table S2B**

ANOVA for response surface quadratic model

| Term | Df | TPC | | TAC | |
| --- | --- | --- | --- | --- | --- |
|  |  | *F* value | *p* value | *F* value | *p* value |
| Mode | 9 | 14.65 | 0.0009^***^ | 7.62 | 0.007^**^ |
| A-Liquid-to-solid ratio | 1 | 9.57 | 0.0175^*^ | 4.55 | 0.0704 |
| B-Water content | 1 | 7.82 | 0.0267^*^ | 4.7 | 0.0667 |
| C-Ultrasound power | 1 | 0.3 | 0.6034 | 4.2 | 0.0795 |
| AB | 1 | 0.4 | 0.5495 | 1.19 | 0.3119 |
| AC | 1 | 2.68 | 0.1455 | 2.77 | 0.1403 |
| BC | 1 | 0.075 | 0.7919 | 1.34 | 0.2848 |
| A^2^ | 1 | 2.29 | 0.1739 | 2.68 | 0.1455 |
| B^2^ | 1 | 96.4 | < 0.0001^***^ | 40.36 | 0.0004^***^ |
| C^2^ | 1 | 6.77 | 0.0353^*^ | 3.62 | 0.0988 |
| R^2^ |  | 0.9496 | | 0.9073 | |
| Adj R^2^ |  | 0.8848 | | 0.7882 | |
| Pre R^2^ |  | 0.5981 | | 0.5846 | |
| Adeq precision |  | 9.506 | | 8.708 | |
| Lack of fit (*F*-value) | 10 | 1.06 | | 2.93 | |
| Lack of fit (*p*-value) |  | 0.4574^ns^ | | 0.1633^ns^ | |

ns, Not significant (*p* > 0.05).

* Significant at (*p* < 0.05).

** Highly significant at (*p* < 0.01).

***Extremely significant at (*p* < 0.001).
